# Positive social relations, loneliness, and immune system gene regulation

**DOI:** 10.1111/nyas.15372

**Published:** 2025-06-01

**Authors:** Sung‐Ha Lee, Jeanyung Chey, Incheol Choi, Yoosik Youm, Steve Cole

**Affiliations:** ^1^ Department of Psychology Yonsei University Seoul South Korea; ^2^ Department of Psychology Seoul National University Seoul South Korea; ^3^ Center for Happiness Studies Seoul National University Seoul South Korea; ^4^ Department of Sociology Yonsei University Seoul South Korea; ^5^ Department of Psychiatry & Biobehavioral Sciences University of California Los Angeles California USA

**Keywords:** CTRA, loneliness, positive relations with others, social orientation

## Abstract

Perceived isolation (i.e., loneliness) has been linked to an immune response gene profile known as the conserved transcriptional response to adversity (CTRA), but little is known about how positive social relations might affect human genome function. We analyzed two studies of Korean adults to determine whether the positive qualities of an individual's general social relations with others (warmth, satisfaction, and trust; as measured by the Positive Relations with Others [PRWO] subscale of the The Ryff Scales of Psychological Well‐being) might be inversely associated with CTRA gene expression. In Study 1 (53 participants, mean age = 72 years, 47% female), PRWO were significantly associated with reduced CTRA profiles, even after controlling for loneliness. Similarly, in Study 2 (152 participants, mean age = 45 years, 50% female), PRWO were significantly associated with reduced CTRA profiles, particularly in the context of higher collectivism. These findings suggest that gene regulatory correlates of social flourishing extend beyond the absence of loneliness, and may contribute to health advantages associated with social well‐being. Loneliness and social flourishing may not simply represent opposite ends of a single continuum but rather function as related yet distinct processes affecting human molecular well‐being.

## Graphical abstract

In two studies of Korean adults, positive social relations—characterized by warmth, satisfaction, and trust—are inversely associated with conserved transcriptional response to adversity (CTRA) gene expression, independent of loneliness. Social flourishing may benefit human molecular well‐being, above and beyond the absence of social isolation or loneliness.

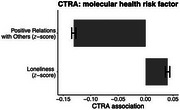

## INTRODUCTION

Social disconnection poses significant threats to human health and well‐being, underscoring the crucial role of social integration in human survival.^1^, ^2^, ^3^, ^4^, ^5^ Social disconnection and isolation have been identified as risk factors for adverse health conditions, such as cardiovascular diseases, mental distress (e.g., depression, anxiety), and all‐cause mortality.^6^, ^7^, ^8^, ^9^, ^10^, ^11^, ^12^ Given its substantial prevalence (e.g., approximately 20%–30% of older adults worldwide experience loneliness^13^), social disconnection emerges as a significant public health issue that necessitates targeted interventions to mitigate it and thereby enhance overall health. Given the health significance of social connection, it is important to understand the mechanisms underlying the link between social disconnection and health. Functional genomic analyses of immune cell gene expression provide one avenue for understanding the biological mechanisms underlying the interplay between social factors and health.

Social genomics is a recently emerging field that analyzes the dynamic interaction of genome regulation with psychosocial environmental factors. The first human social genomic study compared genome‐wide transcriptional profiles of circulating immune cells in chronically lonely individuals with those of socially integrated individuals.^14^ In that study, differential gene expression patterns (greater than 1.3‐fold increase or decrease) were observed in over 100 genes among more than 20,000 gene transcripts surveyed. These distinct gene expression patterns were later found to be prototypical of a conserved transcriptional response to adversity (CTRA)—which is characterized by upregulation of proinflammatory genes and downregulation of antiviral and interferon‐related genes.^15^, ^16^, ^17^ In this framework, social signals, such as loneliness, constitute a survival threat, and thereby activate the body's alert/alarm system via central nervous system social signal transduction pathways. CTRA social signal transduction begins with the psychological process of cognitive appraisal in the brain, which subsequently activates the sympathetic nervous system (SNS) to release norepinephrine. Norepinephrine signals through beta‐adrenergic receptors to activate the transcription factor, cAMP‐response element binding protein (CREB),^18^, ^19^ as well as the proinflammatory NF‐kB transcription factor family, while simultaneously inhibiting the antiviral interferon regulatory factor (IRF) family of transcription factors.^14^, ^20^, ^21^ Such changes in transcription factor activity modulate CTRA gene expression in immune cells and thereby affect disease resistance and immune responses. Since the initial discovery of the links between loneliness and immune cell gene expression patterns, researchers have continued to explore the intricate connections between social experiences and genome biology,^14^ and the associations between loneliness and increased expression of CTRA have been also found in elderly Koreans,^22^ as well as Koreans with higher collectivistic social orientation.^23^

In addition to identifying the body's response to adverse social events like loneliness, social genomics has also begun to explore protective factors that can decrease CTRA expression. These findings have been motivated in part by *social safety* theory, which suggests that positive social processes such as a sense of connection, trust, and inclusion can affect the brain and immune system to promote health and well‐being, potentially counteracting the negative effects of social threat and isolation.^24^ Several other conceptually related psychological resilience factors, including a higher sense of purpose in life, eudaimonic well‐being (happiness derived from meaning and purpose in life), and prosocial behaviors such as acts of kindness toward others, have also been associated with decreased levels of CTRA.^25^, ^26^, ^27^, ^28^, ^29^, ^30^ However, there is little research on whether the positive qualities of an individual's general social relations with others (e.g., warmth, satisfaction, and trust) affect CTRA expression, or whether such effects might be independent of perceived social disconnection/isolation (i.e., loneliness).

In this study, we propose positive social relations as a social connection factor that is related to, but conceptually distinct from social disconnection (loneliness), and examine whether this factor could further regulate CTRA profiles above and beyond the effects of loneliness. Social connection has multifaceted aspects and is typically conceptualized into three categories: structural, functional, and qualitative features.^4^ The CTRA has previously been examined in relation to structural (i.e., social network position)^22^ and functional (i.e., perceived loneliness)^14^, ^23^, ^31^ aspects of social connection. However, the qualitative aspect of social connection has been less studied in the social genomics literature thus far. The quality of social connection serves as one essential element of a good life or flourishing.^32^, ^33^, ^34^ The construct of Positive Relations with Others (PRWO) subscale of the Ryff Scales of Psychological Well‐being was derived from the eudaimonic framework of psychological well‐being^35^, ^36^ and captures relational qualities such as warmth, trust, and satisfaction, which are distinct from structural (e.g., number of friends) or functional (e.g., frequency of social interactions) dimensions of social connection. Maintaining PRWO is one of the components of eudaimonic well‐being—the realization of one's potential and living a life of virtue experiencing warm and satisfying relationships with others. Previous studies suggest that PRWO reflects subjective appraisals of relational quality, capturing dimensions such as warmth, trust, and satisfaction, which have been linked to health outcomes. For example, higher PRWO is associated with decreased levels of inflammatory markers such as interleukin‐6.^37^ Moreover, an enriched social network with active social engagement increases feelings of PRWO and predicts functional capacity and longevity in later life.^38^ Even though PRWO, as a component of social connection, has been investigated in the context of epidemiological health outcomes, the molecular mechanisms of those effects remain largely unexplored.

To address this gap, the present study investigates the association between PRWO and CTRA gene expression in circulating immune cells (blood leukocytes) in two samples of Korean adults. Building on social safety theory, we hypothesize that positive social relationships, as measured by PRWO, are inversely associated with CTRA gene expression (above and beyond the absence of loneliness or social structural deficits). Specifically, the sense of safety derived from warm, trusting social ties may reduce stress‐induced proinflammatory gene activation while enhancing antiviral gene expression. Additionally, we propose that this association is moderated by a collectivistic orientation, as cultural values emphasizing relational interdependence may heighten the salience of social connections in mediating a sense of safety and in shaping biological processes and their downstream effects. Even though one prior study found that PRWO was related to the downregulation of CTRA profiles,^26^ the current study proposes to investigate the association between PRWO and CTRA in Korea—a collectivistic society. Collectivistic cultures, emphasizing group harmony and interdependence, may magnify the health benefits of positive social relationships. We utilized data from two studies of Korean adults to examine the relationship between CTRA gene expression (measured using a standard blood RNA profile) and scores on a psychometric measure of PRWO. Additionally, because it remains unclear if all individuals in a collectivistic society are equally affected by PRWO, we analyzed how social orientation (collectivism vs. individualism) might moderate the relationship between PRWO and CTRA expression.

## METHODS

### Study participants

#### Study 1

Study 1 was obtained from the Korean Social Life, Health and Aging Project (KSHAP), designed to examine social networks, health‐related factors, and aging in older Korean adults.^39^ Among the total of 947 participants who were older than 65, 126 underwent neuropsychological assessments after excluding those with neurological or psychiatric conditions, below 1.5 standard deviations (SDs) of Mini‐Mental State Examination for Dementia Screening (MMSE‐DS scores), or diabetes/hypertension. Of the 126 participants, 85 were followed up in Wave 2 (2021) for medical exams, including blood collection for RNA analysis. Among those 85 samples, three were excluded from analysis due to poor RNA quality, 12 due to recent illnesses, and 17 due to age outliers (>2 SDs above the mean age of 80) leaving a final analyzed dataset of 53 samples (mean age = 72 years ± 4, 47% female).^22^ The Study 1 subsample was younger and had a larger social network size than the whole study cohort (*z* = −2.40, *p* < 0.05 and *z* = 8.33, *p* < 0.001, respectively), likely due to the health inclusion criteria noted above. All participants provided written consent prior to participation, and the study was approved by institutional review boards of Yonsei University and Seoul National University.

#### Study 2

Study 2 was obtained from the Korean Adult Longitudinal Study (KALS). The first wave of KALS included 561 participants aged 20–69 (mean age 44.82 ± 13.83 years; 281 females) from the Seoul metropolitan area. Among the 561 participants in Wave 1 (ages 20–69), a representative subsample was selected for a biomarker substudy, with 33 in their 20s, 29 in their 30s, 32 in their 40s, 32 in their 50s, and 26 in their 60s, balanced by gender; *n* = 152 participants (mean age = 45 ± 13.83 years, 50% female). The Study 2 subsample was representative in terms of age and the scores of PRWO (*z* = 0.17, *p* = 0.86; *z* = 0.68, *p* = 0.49, respectively). The biomarker substudy involved visits to a clinic affiliated with Seoul National University, where participants underwent physical examinations, blood sample collection, and psychological assessments. All participants provided written consent prior to participation, and the Institutional Review Board of Seoul National University and SMG‐SNU Boramae Medical Center approved all procedures and materials.

### Measurements

#### Positive relations with others

PRWO was assessed using a three‐item version of Ryff Scales of Psychological Well‐being as an index of social connection quality.^35^, ^36^, ^40^ The three‐item measure was used due to practical constraints in data collection for both studies (minimizing response burden/length while maximizing correlation with the original 9‐item PRWO subscale). The subscale items included: “Maintaining close relationships has been difficult and frustrating for me” (reverse‐scored), “People would describe me as a giving person, willing to share my time with others,” and “I have not experienced many warm and trusting relationships with others” (reverse‐scored). Response options ranged from 1 = strongly disagree to 5 = strongly agree, and the mean scores were used for analysis. Cronbach's alpha = 0.68, M = 3.85, SD = 0.63 for Study 1 and Cronbach's alpha = 0.61, M = 3.89, SD = 0.66, respectively).

#### Loneliness

Loneliness was assessed with the 20‐item UCLA Loneliness Scale.^41^ Participants rated how often they felt the way they did using a four‐point scale (1 = never, 4 = often; e.g., “How often do you feel that you lack companionship?”; Cronbach's alpha = 0.92, M = 1.80, SD = 0.50 for Study 1 and Cronbach's alpha = 0.88, M = 1.77, SD = 0.47 for Study 2, respectively).

#### Social orientation (used in Study 2 only)

Individuals’ social orientation was assessed with the Individualism–Collectivism scale.^42^ Participants used a nine‐point scale (1 = never, 9 = always) to assess their levels of individualistic orientation (eight items; e.g., “I would rather depend on myself than others”; Cronbach's alpha = 0.74, M = 5.83, SD = 1.03) and collectivistic orientation (eight items; e.g., “If a coworker gets a prize, I would feel proud”; Cronbach's alpha = 0.78, M = 6.52, SD = 0.95). We subtracted individualism scores from collectivism scores to yield a single index of social orientation, such that higher numbers indicate greater collectivism and less individualism (see Ref. 23).

### Blood collection

Blood samples (2.5 mL) were collected into PAXgene RNA tubes and stored at −20°C until analysis. The samples were shipped to the UCLA Social Genomics Core Laboratory, where total RNA was extracted (Qiagen RNeasy), checked for suitable mass, normalized, and subsequently sequenced using a high‐efficiency mRNA‐targeted assay (Lexogen QuantSeq 3′ FWD) conducted on an Illumina NextSeq instrument following the manufacturer's standard protocols, as previously described.^22^, ^23^, ^29^ Sequencing targeted 5 million single‐stranded reads per sample (achieved average = 7.3 million), each of which was mapped to the GRCh38 reference human transcriptome using the STAR aligner (average mapping rate = 99.5%). Gene transcript abundance per million mapped reads was floored at 1 to suppress spurious low‐range variability and log2‐transformed for linear statistical analyses as described below.

### Statistical analysis

The analyses focused on 53 a priori selected gene transcripts utilized to assess the CTRA gene expression profile in previous studies.^23^, ^25^, ^31^, ^43^ Of the 53 genes analyzed, 19 were proinflammatory genes (*IL1A*, *IL1B*, *IL6*, *IL8*, *TNF*, *PTGS1*, *PTGS2*, *FOS*, *FOSB*, *FOSL1*, *FOSL2*, *JUN*, *JUNB*, *JUND*, *NFKB1*, *NFKB2*, *REL*, *RELA*, and *RELB*) and were considered as positive indicators. The remaining 34 genes were involved in type I IFN responses (*GBP1*, *IFI16*, *IFI27*, *IFI27L1‐3*, *FITM4P*, *IFITM5*, *IFNB1*, *IRF2*, *IRF7/8*, *MX1/2*, *OAS1‐3*, and *OASL*) and antibody synthesis (*IGJ*, *IGLL1*, and *IGLL3*), which were sign‐inverted to reflect their inverse contribution to the CTRA. Using SAS PROC MIXED, we examined the association between CTRA (log2‐mRNA levels) and *z*‐score standardized measures of PRWO, loneliness, and social orientation. To control for the correlation among multiple CTRA indicator transcripts within individuals, the 53 CTRA indicator genes were analyzed as repeated measures with a fully parameterized covariance matrix. Consistent with previous studies,^23^, ^25^, ^29^, ^31^ analyses controlled for a standard set of covariates including age, sex, body mass index, health‐related behaviors (e.g., smoking status: current smoker coded as 1 and nonsmoker as 0; alcohol consumption: more than once a week coded as 1 and less as 0), and illness symptoms (e.g., headache, upset stomach) reported during the past month. To further explore interaction effects, we included continuous interaction terms (i.e., social orientation × PRWO) in the mixed linear model. To help visualize the nature of these interactions, we conducted follow‐up simple slopes analyses quantifying the effect of PRWO on CTRA gene expression at the mean level of social orientation, as well as at one SD above and below the mean social orientation. Parameter estimates with standard errors and 95% confidence intervals (CIs) were reported, and *p* values <0.05 were considered statistically significant.

As sensitivity analyses, we repeated our main analyses after further adjusting for socioeconomic status (SES) or structural social network, social support, chronic diseases conditions (Study 1 only), and purpose in life as measured by three items from Ryff Psychological Well‐being Scale.

## RESULTS

### Study 1

**FIGURE 1:**
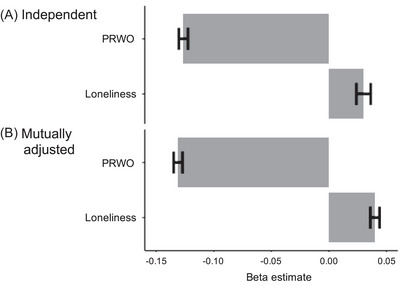
Positive relations with others (PRWO)/loneliness and conserved transcriptional response to adversity (CTRA) gene expression. (A) Separate analysis and (B) mutually adjusted (each was adjusted each other with covariates).

Loneliness and PRWO showed a significant inverse association, *r* = −0.77, *p* < 0.001, as expected. CTRA gene expression also showed a significant inverse relationship with PRWO (*b* = −0.126 log2 mRNA/SD PRWO, 95% CI [−0.134, −0.119], SE = 0.004, *t*(46) = −31.55, *p* < 0.001; Figure 1). This association remained significant after controlling for the degree of loneliness (*b* = −0.131, 95% CI [−0.139, −0.123], SE = 0.004, *t*(45) = −33.88, *p* < 0.001). The findings also remained significant in sensitivity analyses that additionally adjusted for SES, structural social network, social support, chronic disease conditions, or purpose in life (Table S1).

### Study 2

Study 2 examined participants from a metropolitan area with a wider age range. PRWO was again inversely associated with loneliness (*r* = −0.69, *p* < 0.001). When analyzing the general association between PRWO and CTRA, no significant relationship was found (*b* = −0.008, 95% CI [−0.022, 0.006], SE = 0.007, *t*(144) = −1.15, *p* = 0.253). However, considering the significant effect of social orientation in moderating the relationship between loneliness and CTRA in previous research on this sample,^23^ we also considered whether collectivism versus individualism might moderate the relationship between CTRA and PRWO. With the inclusion of that interaction term, the marginal effect of PRWO was statistically significant (*b* = −0.024, 95% CI [−0.047, −0.001], SE = 0.012, *t*(142) = −2.08, *p* = 0.034), and the marginal effect of collectivistic social orientation also showed significance (*b* = 0.157, 95% CI [0.073, 0.241], SE = 0.043, *t*(142) = 3.62, *p* < 0.001). Importantly, these effects were qualified by a significant interaction between PRWO and social orientation (*b* = −0.034, 95% CI [−0.056, −0.012], SE = 0.011, *t*(141) = −3.11, *p* = 0.002) such that the inverse association of CTRA with PRWO was more pronounced among those with higher collectivist social orientation. These findings were generally consistent in the sensitivity analyses when additionally adjusting for SES, structural social network, social support, or purpose in life (Table S2).

**FIGURE 2:**
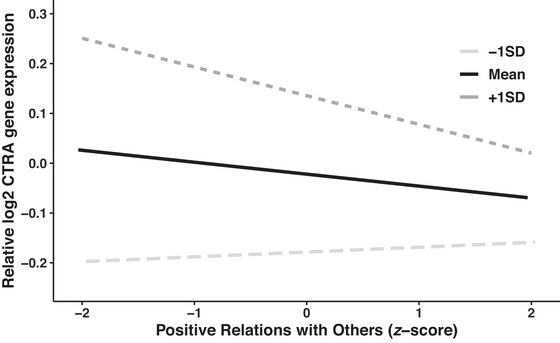
The association between positive relations with others and CTRA, moderated by social orientation with collectivism (vs. individualism); the dark gray line indicates a higher degree of collectivism compared to individualism.

To understand this interaction effect, we conducted follow‐up simple slopes analyses to examine the effect of PRWO for Korean adults who are more collectivistic (one SD above the mean of the social orientation index) and for those who are less collectivistic (one SD below the mean) separately. As depicted in Figure 2, PRWO was significantly associated with decreased levels of CTRA gene expression among those who are more collectivistic (*b* = −0.058, 95% CI [−0.095, −0.021], SE = 0.019, *t*(142) = −3.01, *p* = 0.003). In contrast, there was no relationship between PRWO and CTRA gene expression among those who are less collectivistic (*b* = 0.010, 95% CI [−0.021, 0.041], SE = 0.016, *t*(142) = 0.610, *p* = 0.543).

## DISCUSSION

In the present study, we investigated the relationship between positive social relationship quality and CTRA gene expression in two cohorts of Korean adults. In the first study, involving older adults over the age of 65 from a rural area, those who perceived their general social relationships as positive, warm, and supportive exhibited significant downregulation of CTRA profiles. The observed effect sizes correspond to an approximately 30% difference in CTRA mRNA abundance across the range of a continuous (*z*‐score) measure of PRWO. Moreover, these effects of positive social relationship quality emerged above and beyond the effects of low perceived social isolation (loneliness), suggesting that these two aspects of social well‐being are at least partially distinct influences on CTRA gene regulation.

In a second study with a broader age group from an urban setting, we partially confirmed the link between positive relationships and CTRA downregulation, particularly among individuals with a higher collectivistic social orientation (who showed approximately 15% difference in CTRA mRNA across their general range of variation in PRWO). In both studies, we utilized the PRWO measure, a component of psychological (eudaimonic) well‐being, to assess the quality of social connections, providing insights into how individuals flourish through warm relationships while also characterizing the quality dimension of social connections beyond previously analyzed dimensions of structure and function.^4^ When we further controlled the effect of structural aspects of social network for Studies 1 and 2, PRWO continued to show inverse associations with CTRA. As such, PRWO appears to capture unique aspects of social connections beyond structural features. The current results align with prior findings that emphasize the health advantages of positive ties with close ones, such as family,^44^, ^45^ which focus on the quality of social relationships. Additionally, the findings are consistent with the health impact of psychological well‐being identified in several previous studies.^25^, ^29^, ^33^ Moreover, since this association extends beyond the mere absence of loneliness, loneliness and positive social relations may not simply represent opposite ends of one spectrum but rather function as related yet distinct processes that affect health.

The current findings suggest a link between the perception of safe and warm relationships and the downregulation of the CTRA gene expression profile in the immune system. Our findings align with social safety theory, which hypothesizes that positive social relationships provide a sense of safety that mitigates the body's stress–response system, leading to reduced CTRA activation. This biological process, connecting psychosocial factors to immune gene expression patterns, involves multiple steps from brain social appraisal through SNS activity to consequent downstream immune cell signal transduction.^15^, ^18^, ^46^ Among the possible physiological mediators, it is plausible that the parasympathetic nervous system, particularly vagus nerve activity, might play a role. This speculation is supported by recent studies that have provided empirical evidence linking increased parasympathetic function to reduced CTRA gene expression patterns.^47^, ^48^ Specifically, increased vagus nerve activity, as measured by heart rate variability, has been linked to reduced NF‐κB activity and increased IRF activity. Interestingly, the vagus nerve is also hypothesized to play an important role in forming social relationships, as proposed by the polyvagal theory.^49^, ^50^ The polyvagal theory proposes that the ventral vagus nerve is heavily engaged in forming and maintaining safe social relationships, in contrast to the fight‐or‐flight system. Consequently, individuals experiencing safe relationships may exhibit increased tonic vagus nerve activity, which could downregulate sympathetic nervous system‐driven CTRA gene expression as a result.

The current findings support the association between warm relationships with others and immune‐related gene expression. This relationship was particularly pronounced among older adults in Study 1 and younger individuals with higher collectivistic tendencies in Study 2. Given the heterogeneous, multifaceted, and complex nature of individual social contexts, which can be interpreted in various ways, it is essential to investigate the dynamic factors that moderate the relationship between social connection and health outcomes. For example, a recent study explored the role of social motivation (e.g., social approach and social avoidance) in regulating the link between social isolation and CTRA profiles.^51^ Moreover, while prior studies have examined associations between CTRA gene expression and PRWO or related measures of social well‐being,^26^, ^30^ our study extends those findings by considering the role of loneliness, examining relationships in a collectivistic cultural context (East Asian), and further exploring moderation by collectivistic social orientation. The present findings highlight the importance of considering individual variations in social orientation when investigating the links between positive social relations and immune gene expression.

This moderation of PRWO effects by collectivistic orientation is consistent with prior studies showing that a collectivistic cultural orientation, which motivates individuals to fit in with others and prioritize group membership, can be detrimental to well‐being.^52^ This may be because collectivistic individuals perceive themselves as deeply interconnected with others, making their health and well‐being heavily reliant on maintaining social harmony.^53^, ^54^ The significant moderation of collectivistic orientation underscores the role of cultural values in shaping the physiological impact of social relationships. Given that social orientation does not significantly correlate with health or demographic factors such as age in this sample (Table S3), the moderating role of collectivism in the PRWO–CTRA relationship appears to be general in this context. However, we also recognize that our findings are specific to Korea, and further cross‐cultural research is needed to test whether similar associations hold in individualistic or other collectivistic cultural contexts. So far, the relationship between cultural orientation and health and well‐being has shown mixed results; for example, a positive relationship between collectivism and happiness^55^ but a negative association between collectivism and life satisfaction in some contexts.^56^ Because studies on the link between physical outcomes and cultural orientation are limited, future research should examine when and where cultural norms can amplify or mitigate social threats that might potentially activate CTRA pathways.

There are several limitations to be noted. First, although we propose social/cultural orientation as a possible moderator that influences individual differences in social quality and health benefits, Study 1 was missing this variable. Given that participants of Study 1 were all elderly adults living in an agrarian, rural society possibly with Confucianism and traditional East Asian values, we can likely assume that they show a stronger tendency toward collectivism than younger and more modernized generations, based on the results from prior studies.^57^, ^58^ Future study designs incorporating direct measures of collectivism are needed to explicitly confirm the relationship between social orientation, social connection, and CTRA profiles. Second, as the present study targeted Korean adults, our findings should be interpreted cautiously when generalizing to other countries and cultures. The present findings are based on cross‐sectional associations, which cannot establish causal relationships. Future studies with longitudinal designs or experimental manipulations of social conditions will be necessary to determine the temporal (bi)directionality of social connection and CTRA gene expression patterns. Fourth, to connect CTRA profiles with physical outcomes such as chronic diseases, which are related to social disconnection, it would be ideal to combine other clinical measures (e.g., blood pressure or CT scans) with longitudinal tracking. This approach would help elucidate the biological mechanisms linking social connection to physical health. Moreover, even though we controlled for key covariates that could impact CTRA profiles, the binary coding of some of these factors (e.g., alcohol consumption as “more than once a week” vs. “less than once a week” and smoking status as “current smoker” vs. “nonsmoker”) may limit precision by failing to capture nuanced patterns of behavior. Future studies should employ more detailed and comprehensive measures for these and other covariates to better assess their potential impact. We also acknowledge the potential for selection bias given that biomarker data were only available for a subset of participants. This issue is particularly relevant for Study 1, where the subsample was significantly younger and better educated than the overall study population. Healthy subset bias may limit the generalizability of our findings, which should be further evaluated in future research with larger samples.

We also cannot exclude the possibility of unmeasured confounders, such as prosocial behavior. Future studies should aim to include larger and more representative samples to enhance the generalizability of the findings. Also, while our study emphasizes the quality of social relationships, prior research indicates that even those with high‐quality connections may remain at risk for adverse health outcomes if they lack sufficient structural and functional support.^59^, ^60^ Although our analyses do not find structural social factors accounting for the PRWO–CTRA relationship, other unexamined factors may contribute. Future research with broader covariate adjustments and longitudinal designs is needed to confirm these findings and rule out alternative explanations. Lastly, the abbreviated version of the PRWO scale used in this study may not fully capture the construct of relationship quality (i.e., relative to the full nine‐item PRWO subscale of the Ryff well‐being measure or other broader measures of social relational quality). Moreover, even this limited three‐item measure is conceptually complex, and may also encompass closely related positive social processes such as prosocial tendencies or secure attachment styles, rather than solely measuring relational satisfaction and trust. As the construct of PRWO is conceptually complex, the observed associations between PRWO and CTRA expression may be at least in part attributed to prosocial behaviors, which have been independently linked to CTRA activity and immune function.^27^, ^28^ Future research should consider employing the full nine‐item PRWO scale or other validated and comprehensive measures of relationship quality to provide a more nuanced understanding of these associations.

## CONCLUSION

Despite these limitations, the current findings suggest that maintaining high‐quality social relationships with others may be one positive psychosocial factor that can promote molecular well‐being and human health. Future studies examining neural mechanisms (e.g., parasympathetic function) and more extensive measures of social factors (e.g., functional and structure aspects) will further elucidate the mechanisms involved and suggest new interventions to promote human health in its inherently social context.

## AUTHOR CONTRIBUTIONS

Sung‐Ha Lee and Steve Cole conceptualized the work and conducted the formal analysis. Sung‐Ha Lee wrote the original draft, and Steve Cole supervised the project and reviewed and edited the final draft. Jeanyung Chey, Incheol Choi, and Yoosik Youm acquired funding and provided supervision. All the authors approved the final submitted version.

## CONFLICT OF INTEREST STATEMENT

The authors declare no conflicts of interest.

## PEER REVIEW

The peer review history for this article is available at https://publons.com/publon/10.1111/nyas.15372.

## Supporting information



Supporting Information

## Data Availability

All data, analysis code, survey materials, and supporting information are available upon request from the authors.
