# Supplementary material for: Positive social relations, loneliness, and immune system gene regulation
Source: Ann N Y Acad Sci. 2025 Jun 1;1549(1):139–47. doi: 10.1111/nyas.15372 (PMC12309429; doi:10.1111/nyas.15372)
Supplement: Supplementary file 1 — Supporting Information [file NYAS-1549-139-s001.docx]

Supporting Table S1. Sensitivity analyses of the association between PRWO and CTRA profiles in Study 1.

|  | Estimate | SE | DF | t value | p value |
| --- | --- | --- | --- | --- | --- |
| Original covariates^1^ | -0.1264 | 0.004 | 46 | -31.55 | < .0001 |
| + strucutral social network^2^ | -0.1225 | 0.004 | 45 | -31.05 | <.0001 |
| + SES^3^ | -0.0731 | 0.004 | 45 | -18.94 | <.0001 |
| + chronic disesases^4^ | -0.1142 | 0.003 | 45 | -36.46 | <.0001 |
| + purpose in life^5^ | -0.1691 | 0.004 | 45 | -46.07 | <.0001 |
| +social support^6^ | -0.1371 | 0.004 | 45 | -34.76 | <.0001 |

^1^ All results control for age, gender, BMI, smoking, and alcohol consumption.

^2^ Structural social network refers to network size, summing up the spouse and discussion network members. Marital status was not included, as 49 out of 53 participants were married. However, controlling for marital status (coded as not married = 0, married = 1) still resulted in a significant PRWO effect (*b* = -0.1350, *SE* = 0.003, *t*(45) = -48.17, *p* < 0.001)

^3^ SES (subjective socioeconomic status) was coded on a scale from 1 (lower bottom) to 6 (upper top)

^4^ Chronic diseases include diabetes, hypertension, or dyslipidemia. Participants with any of these conditions were coded as 1, and those without were coded as 0

^5^ Purpose in life was measured using three items from the Ryff Psychological Well-being Scale

^6^ Social support was measured using the averaging response to the question “How often can you rely on the network member for help if you have a problem?”, rated on a scale from 1(never) to 4 (often)

Supporting Table S2. Sensitivity analyses of the interaction between PRWO and social orientation in relation to CTRA profiles in Study 2.

|  | Estimate | SE | DF | t value | p value |
| --- | --- | --- | --- | --- | --- |
| Original covariates^1^ | -0.0337 | 0.011 | 142 | -3.11 | 0.0023 |
| + strucutral social network^2^ | -0.0395 | 0.011 | 135 | -3.59 | 0.0005 |
| + SES^3^ | -0.0359 | 0.011 | 141 | -3.30 | 0.0012 |
| + purpose in life^4^ | -0.0325 | 0.011 | 141 | -3.02 | 0.0030 |
| +social support^5^ | -0.0345 | 0.011 | 141 | -3.19 | 0.0018 |

^1^ All results control for age, gender, BMI, smoking, and alcohol consumption.

^2^ Structural social network includes the following factors: marital status (0 = not married, N = 53; 1 = married, N = 98), number of close friends ("How many close friends do you have?"), and meeting frequency ("On average, how often do you meet your friends per week?")

^3^ SES (subjective socioeconomic status) was coded on a scale from 1 (lower bottom) to 10 (upper top)

^4^ Purpose in life was measured using three items from the Ryff Psychological Well-being Scale

^5^ Social support was measured using the Multidimensional Scale of Perceived Social Support^1^

Supporting Table S3. Correlations between social orientation, demographic, and health factors in Study 2

| Variable | *M* | *SD* | 1 | 2 | 3 | 4 | 5 |
| --- | --- | --- | --- | --- | --- | --- | --- |
| 1. Social orientation | 0.69 | 1.24 |  |  |  |  |  |
| 2. age | 44.64 | 14.02 | .14 |  |  |  |  |
|  |  |  | [-.02, .30] |  |  |  |  |
| 3. BMI | 23.91 | 3.84 | .15 | .08 |  |  |  |
|  |  |  | [-.00, .31] | [-.08, .23] |  |  |  |
| 4. alcohol consumption | 0.22 | 0.42 | -.08 | -.24** | -.05 |  |  |
|  |  |  | [-.23, .08] | [-.38, -.08] | [-.21, .11] |  |  |
| 5. smoking | 0.39 | 0.49 | .02 | -.00 | .14 | .29** |  |
|  |  |  | [-.14, .18] | [-.16, .15] | [-.02, .29] | [.13, .43] |  |
| 6. illness symptom | 2.68 | 1.74 | -.15 | -.15 | -.02 | .18* | .04 |
|  |  |  | [-.30, .01] | [-.31, .01] | [-.18, .14] | [.02, .33] | [-.12, .20] |

References)

1. Zimet, G. D., Dahlem, N. W., Zimet, S. G. & Farley, G. K. The Multidimensional Scale of Perceived Social Support. *J. Pers. Assess.* **52**, 30–41 (1988).
